# Frailty Trajectories and Social Determinants of Health of Older Adults in Rural and Urban Areas in the U.S.

**DOI:** 10.3390/jal5030027

**Published:** 2025-08-08

**Authors:** Hillary B. Spangler, David H. Lynch, Wenyi Xie, Nina Daneshvar, Haiyi Chen, Feng-Chang Lin, Elizabeth Vásquez, John A. Batsis

**Affiliations:** 1 Division of Geriatric Medicine, UNC School of Medicine, Chapel Hill, NC 27599, USA; 2 Department of Biostatistics, Gillings School of Global Public Health, University of North Carolina at Chapel Hill, Chapel Hill, NC 27599, USA; 3 Department of Epidemiology and Biostatistics, College of Integrated Health Science, University at Albany, Albany, NY 12222, USA; 4 Carolina Population Center, University of North Carolina at Chapel Hill, Chapel Hill, NC 27516, USA; 5 Department of Nutrition, Gillings School of Global Public Health, University of North Carolina at Chapel Hill, Chapel Hill, NC 27599, USA

**Keywords:** frailty, frailty trajectory, social determinants of health, geriatrics, mortality, skilled nursing, rural

## Abstract

Older adults, aged 65 years and older, develop and experience frailty at different rates. Yet, this heterogeneity is not well understood, nor are the factors, such as geographical residence, that influence different frailty trajectories and subsequent healthcare outcomes. We aim to identify factors that impact older adult frailty trajectories, skilled nursing facility (SNF) placement, and death. Medicare beneficiaries ≥ 65 years from the National Health and Aging Trend Study (2011–2021) with complete data using Fried’s frailty phenotype on ≥ 2 occasions (*n* = 6082) were included in the analysis. Rural/urban residence was defined using Office of Management and Budget criteria. Latent class growth analysis (LCGA) helped identify four frailty trajectories: improving, stable, mildly worsening, and drastically worsening. Cox proportional hazard analysis and logistic regression determined the association of social determinants of health (sex, race/ethnicity, education and income level, healthcare and transportation access, and social support) on death and SNF admission, respectively. The mean age was 75.12 years (SE 0.10); 56.4% female, 18.6% (*n* = 1133) rural residence. In the overall sample, 1094 (23.0%) older adults were classified as robust, 3242 (53.0%) as pre-frail, and 1746 (24.0%) as frail. Urban residence did not modify the relationship between frailty trajectories and SNF placement, nor did geographic residence on death. Higher income was associated with lower odds of a worse frailty trajectory, SNF admission, and a lower hazard of death, all reaching statistical significance. Future work should examine the factors that influence older adult participation in research and the impact of standardizing the definition of geographic rurality on older adult frailty and health outcomes.

## 1. Introduction

Frailty is a geriatric syndrome associated with aging, as older adults are at an increased risk of physical vulnerability as they age [1]. Older adults with frailty, especially in rural areas, face heightened risks of morbidity and mortality in rural areas, including increased rates of hospitalization and placement in skilled nursing facilities (SNFs) [2-4]. The risk of worse healthcare outcomes in rural areas may be influenced by social determinants of health (SDOH) characteristics, such as lower income levels, lower education levels, and limited access to healthcare resources and supportive communities [5-8]. The heterogeneity of frailty complicates the delivery of individualized treatments for frailty mitigation, as clinical interventions have historically lacked an individualized approach.

Frailty is a dynamic syndrome, transitioning between frailty, pre-frailty, and robustness [9]. Gill et al. observed transitions in frailty states of community-dwelling older adults over time [9]. These dynamic frailty transitions pose opportunities to develop interventions to prevent worsening frailty trajectories. Identifying older adults most at risk for worsening frailty trajectories lends to precision medicine: delivering tailored treatments rather than a “one-size-fits-all” approach [10]. Precision medicine interventions for frailty have not routinely included elements targeting specific SDOH, which can impact downstream healthcare outcomes. Understanding the factors influencing frailty transitions, especially related to SDOH and geographical factors, is important for addressing these gaps in healthcare at both an individual and a system level [5]. Therefore, we aim to use latent growth class analysis to identify characteristics of older adults who are most at risk of worsening frailty trajectories and how this risk is modified by the interaction between SDOH and geographical residence.

## 2. Materials and Methods

### 2.1. Study Participants

We included older adults, aged 65 years and older, from 2011–2021 of the National Health and Aging Trends Survey (NHATS). NHATS is a nationally representative longitudinal cohort of Medicare beneficiaries (*n* = 8245) [11]. Additional information about the NHATS recruitment procedures can be found in the standardized NHATS protocol [12]. Community-dwelling older adults with complete frailty classification data with at least two time points from the 2011 cohort were included, with a possibility of completing eleven survey rounds. Individuals residing in a nursing home or residential care in 2011 were excluded as these individuals typically receive more support than the average community-dwelling older adult. The study received approval from the institutional review board at the University of North Carolina at Chapel Hill (IRB 23-1085).

### 2.2. Residential Status

NHATS classifies metropolitan and non-metropolitan status by 2013 Rural-Urban Continuum Codes for binary metropolitan (3 codes) and non-metropolitan (6 codes) [13]. For this study, metropolitan areas were considered urban, and non-metropolitan areas were considered rural [14,15]. Geographical category was defined using residential location in 2011.

### 2.3. Frailty Phenotypes

Fried’s frailty phenotype was used to categorize frailty phenotypes in each round of the study [1]. Scoring was based on the collection of the following characteristics as one point for each characteristic: exhaustion, low physical activity, weakness, slowness, and unintentional weight loss. Frailty classifications were based on the following scoring: robust (no items), pre-frail (1 or 2 items), and frail (≥3 items). Frailty progression was defined as the accumulation of frailty deficit count using Fried’s phenotype and the respective category. Individuals with only 0 or 1 frailty criteria were excluded if there was only one year of data available.

### 2.4. Skilled Nursing Facility (SNF) Placement, Mortality, and Healthcare Access

We defined skilled nursing facility (SNF) placement as any year in which residential status changed to a SNF, excluding 2011. Mortality (e.g., time to death) was defined as duration in study until death. Lastly, for healthcare access, we included the following binary variables: receiving transportation assistance to the medical visit, having a primary doctor, and seeing a doctor within the last year (in-person or virtual).

### 2.5. Covariates

We included the following self-reported demographic variables: race, ethnicity, sex, employment status, education level, income level, marital status, and smoking status. We also included insurance payer (in addition to Medicare, if applicable) and multimorbidity (two or more self-reported comorbidities out of a list of nine chronic diseases) (Table A1). We considered sex, race/ethnicity, education and income level, healthcare and transportation access, and social support to represent SDOH.

### 2.6. Statistical Methods

Descriptive statistics including means, standard errors, frequencies, and percentages were used to summarize demographic variables when appropriate, including survey weights provided by NHATS. The comparison between urban and rural was based on two-sample *t*-tests for continuous variables and chi-square tests for categorical variables. Our study used latent class growth analysis (LCGA) to determine frailty trajectories from 2011 (visit 1) to 2021 (visit 11). The trajectories were modelled using β-spline basis functions that produced nonlinear curves. The number of classes was determined by the best model fit using multiple information criteria, as well as the number of participants in each group and interpretability. Class membership was determined by the highest posterior probability after estimating model parameters. Univariate associations between class membership and covariates were determined by analysis of variance (ANOVA) and chi-square tests when appropriate. A multinomial logistic regression was then used to explore the multivariable association, including those covariates that are significant in the univariate analysis. Odds ratios (OR) and their 95% confidence intervals (CI) were reported to show the adjusted tendency of a covariate to be in a particular trajectory group to the reference one. We utilized machine learning algorithms to predict frailty trajectories with four approaches: random forest, support vector machine (SVM), *k* nearest neighborhood (KNN), and XGBoost using R 4.3.3 (Vienna, Austria) [16-19].

The micro-averaged area under the curve (AUC) of the multi-class receiver operating curve (ROC) was used to compare the machine learning algorithm’s prediction performance. Other test performance characteristics, such as sensitivity and specificity, were also considered. A normalized importance score of 100 (unitless) was reported to show the variable importance in predicting the trajectory membership. A variable with a score closer to 100 indicates a better predictor. Finally, we compared survival rates between trajectory groups using the Cox proportional hazards model, including geographical residence as an effect modifier. A hazard ratio (HR) and its 95% CI were reported to indicate statistical significance. All statistical analyses were conducted using SAS 9.4 (Cary, NC, USA) and R 4.3.3 (Vienna, Austria). A *p*-value less than 0.05 or 95% CI of OR and HR not covering one was considered statistically significant.

## 3. Results

Table 1 provides descriptive statistics for the overall cohort (*n* = 6082) with a mean age of 75.12 years (SE 0.10) and 56.4% of the sample being female. Among the participants, 1094 (23.0%) were classified as robust, 3242 (53.0%) were pre-frail, and 1746 (24.0%) were frail. Approximately one-fifth of the sample (*n* = 1133, 18.6%) reported living in a rural residence and 81.2% of participants identified as white. There was no significant difference in frailty classifications between individuals in rural and urban areas (*p* = 0.73). However, there were significant differences in level of education (*p* = 0.02) and income (*p* = 0.02) between rural and urban areas, with those in urban areas generally having both higher income and education levels than those in rural areas. There was no significance in household size between groups. Table A1 shows the characteristics as classified by urban and rural residence.

**Figure 1.**
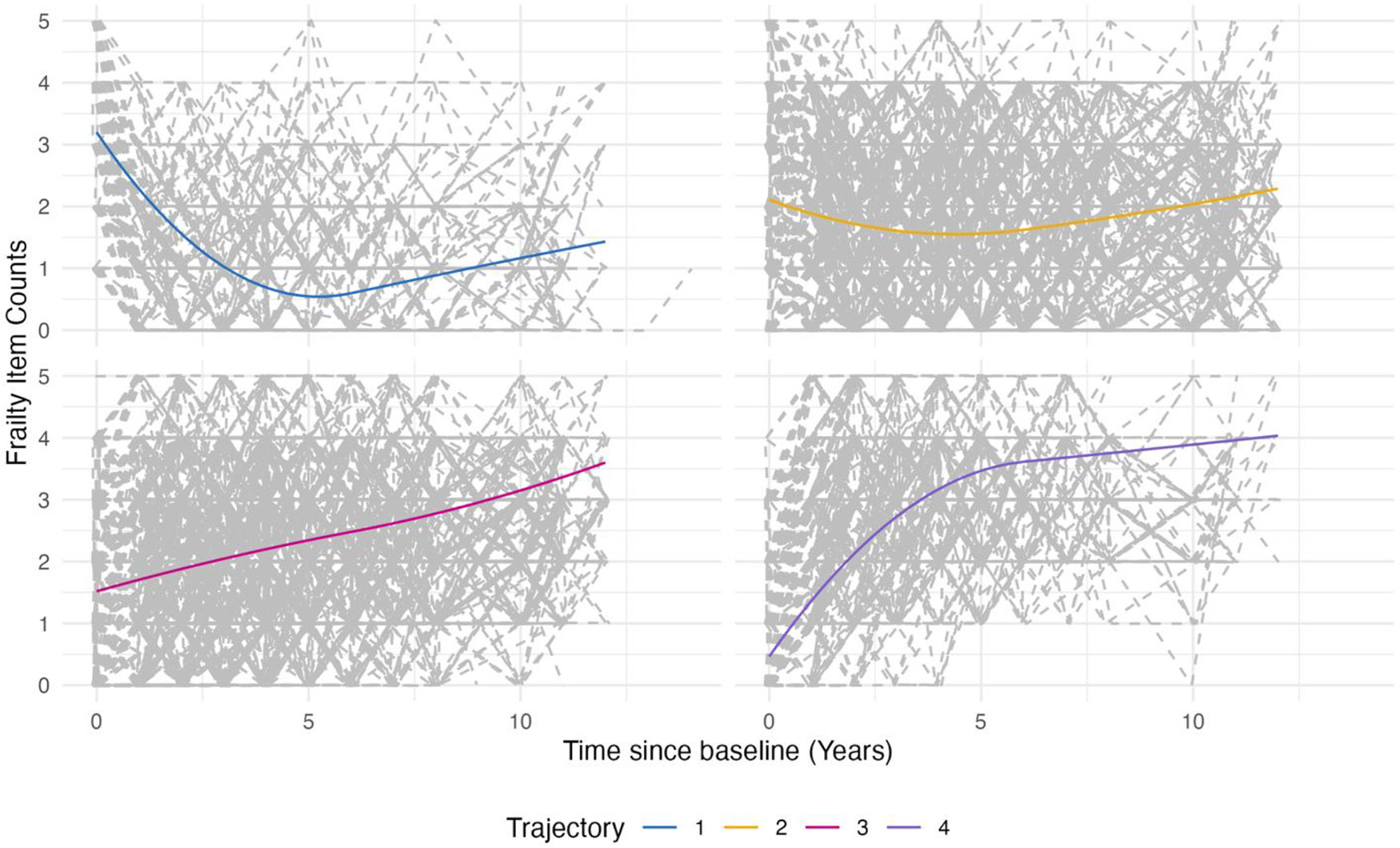
Latent growth class trajectories of frailty counts. Latent growth class trajectories (1 = improving, 2 = stable, 3 = mildly worsening, 4 = drastically worsening) using frailty counts (0–5), over 11 years.

Using latent class growth analysis, we identified four trajectory groups: improving (*n* = 422), stable (*n* = 2396), mildly worsening (*n* = 2518), and drastically worsening (*n* = 746) (Table 1). Table 1 contains the characteristics and descriptive statistics for the four latent classes. Rural or urban residence had no impact on class membership (*p* = 0.17). Classes differed by age group (*p* < 0.001), education level (*p* < 0.001), marital status (*p* < 0.001), income (*p* = 0.03), having insurance in addition to Medicare (*p* < 0.001), needing help with transportation to the doctor (*p* < 0.001), and multimorbidity (*p* < 0.001). A greater proportion of individuals in the mildly and drastically worsening trajectories reported lower income level (USD 0–24,999) and widowed marital status than in the stable and improving classes (Table 1). Additionally, there were fewer participants with a college education and above in the mildly (33.3%) and drastically worsening (28.9%) classes than those with stable (0.0%) or improving (41.5%) trajectories. As shown in Figure 1, the improving class had an initial improvement in frailty status (decreasing frailty counts) until year 5, when frailty counts nadired and then, began to increase. The drastically worsening group had an initial worsening (increased frailty counts) in the first 5 years.

Table 3 shows the multinomial logistic regression results for each latent class with a stable trajectory as the reference category. Older age and English as the primary language were associated with higher odds of being in a worsening frailty trajectories and lower odds of an improving trajectory. Having higher education (mildly, OR = 0.76, 95% CI = 0.63 to 0.91; drastically, OR = 0.64, 95% CI =0.49 to 0.84) and a regular doctor (mildly, OR = 0.81, 95% CI = 0.56 to 1.18; drastically, OR = 0.61, 95% CI= 0.38 to 0.97) were associated with lower odds of worsening frailty trajectories. Needing help with transportation to the doctor was associated with higher odds of mildly (OR = 1.15, 95% CI = 1.01 to 1.32) and drastically worsening (OR = 1.22, 95% CI = 1.00 to 1.48) frailty trajectories.

Table A2 shows the four machine learning models used to predict latent class membership: random forest, SVM, KNN, and XGBoost. Among these, the SVM method performed best, with the greatest area under the curve (AUC = 0.779, 95% CI = 0.774 to 0.787), with an accuracy of 0.757 and sensitivity and specificity of 0.515 and 0.838, respectively, in Table A3). The random forest, KNN, and XGBoost methods performed less optimally with decreasing AUC in corresponding order. For KNN, the tuning parameter of the number of neighbors is K = 83. For SVM, the radial kernel was used, and the tuned hyperparameters are sigma = 1 × 10^−5^ and C = 0.1. For XGBoost, the tuned hyperparameters are nrounds = 600, eta = 0.3, max_depth = 5, gamma = 0.25, colsample_bytree = 0.5, subsample = 0.9, and min_child_weight = 0.2.

### 3.1. Variable Importance

Using a normalized importance score of 100 (e.g., 100 = most important, 0 = least important), we identified age as the most influential predictor (65), with other SDOH with the following impact: income (31.26), education (23.75), household size (21.83), marital status (18.81), and additional insurance to Medicare (18.02). Frailty count and residential status had a lower impact at 29.47, and residential status (rural versus urban) was even lower at 4.78.

### 3.2. Skilled Nursing Home Placement

Table 4 presents the odds of SNF placement for older adults with certain SDOH and latent class membership. Higher frailty counts were associated with higher odds of 1.51 (95% CI = 1.35 to 1.69) of SNF placement. Female sex was associated with higher odds (HR = 1.42, 95% CI = 1.07, 1.90) of SNF placement. Compared to the stable trajectory, the improving trajectory was associated with lower odds of SNF placement. The mildly and drastically worsening trajectories were associated with higher odds of SNF placement, though not reaching statistical significance. Older age (80 years and older), female sex, and lower income brackets were associated with higher odds of SNF placement. Geographical residence did not significantly modify the relationship between latent class membership and SNF placement.

### 3.3. Cox Survival Analysis

**Figure A1.**
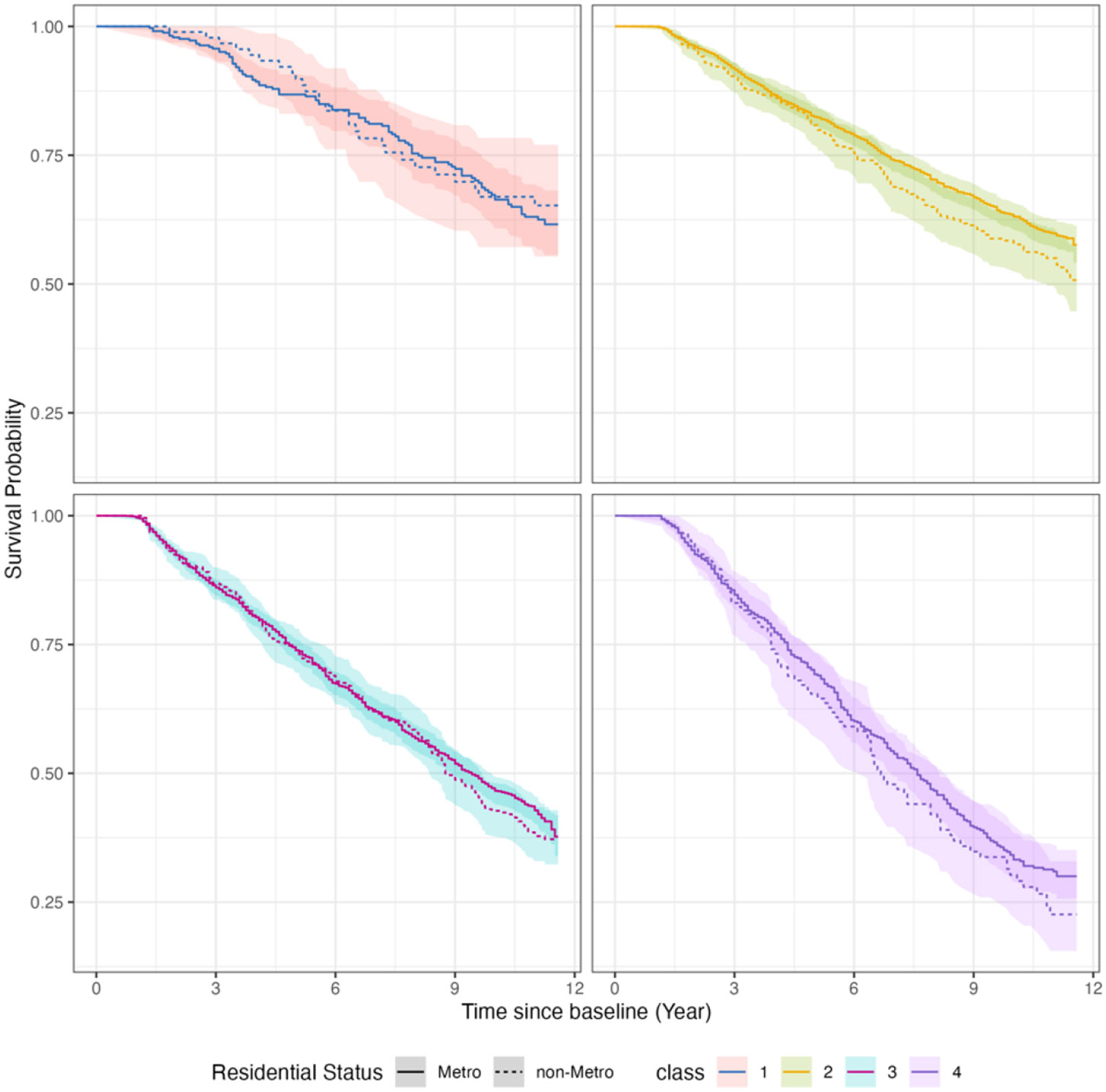
Survival probability by latent classes and residential status. Key: Class 1 = Improving; Class 2 = Stable; Class 3 = Mildly Worsening; Class 4 = Drastically Worsening; Solid line = Urban; Dotted line = Rural.

Table 5 and Figure A1 show the Cox-proportional hazard analysis for mortality of older adults with baseline SDOH and latent class membership. Female sex was associated with lower hazard of death (HR = 0.66, 95% CI = 0.61 to 0.72). The improving frailty trajectory was associated with a lower hazard ratio of mortality (HR = 0.54, 95% CI = 0.36 to 0.82) compared to the stable trajectory. Meanwhile, the mildly (HR = 1.7, 95% CI = 1.38 to 2.09) and drastically worsening (HR = 3.01, 95% CI= 2.30 to 2.09) trajectories were associated with a higher hazard of death when compared with the stable trajectory. For all trajectory classes, hazard ratios were close to 1 when including the urban modifier. Higher frailty counts and older age were associated with higher mortality.

## 4. Discussion

In this nationally representative sample, SDOH—but not rurality—were associated with frailty progression and worse health outcomes. Older adults with improving frailty trajectories had less risk of SNF placement and death, and those with mildly and drastically worsening frailty trajectories had a higher risk of SNF placement and death. Identifying and addressing SDOH that influence frailty trajectories and healthcare outcomes has the potential to mitigate frailty, reduce SNF placement, and lower mortality.

Several SDOH had a significant relationship with class membership, such as education, income, support networks, healthcare access, and race/ethnicity. Older adults with higher levels of education (college and beyond) had a greater proportion with improving and stable frailty trajectories, with “college and beyond” having lower odds of mildly or drastically worsening frailty trajectories (Table 3). Similarly, higher income brackets were associated with higher representation in the stable and improving trajectories and lower odds of worsening trajectories (Table 3). Our findings are consistent with previous literature suggesting that individuals with higher education and income levels facilitate access to financial resources and health literacy, which may contribute to more favorable frailty trajectories [20].

Though marital status was not significantly associated with frailty trajectory class, our findings suggest a possible role for community/relational support in the mitigation of worsening trajectories. A higher percentage of married older adults was represented in the stable and improving frailty trajectories, whereas higher percentages of widowed older adults were represented in the mildly and drastically worsening frailty trajectories (Table 1). The improving frailty trajectory had the lowest proportion of individuals in the widowed, never married, and living with partner categories. Our findings align with previous studies discussing “social frailty,” where older adults with stronger support systems may be less likely to experience frailty than those without a stable support systems (e.g., single status) [20-22]. While limited by a small sample size of older adults identifying as Hispanic race/ethnicity, the lower odds of SNF placement and hazard of death for older adults identifying as Hispanic may be attributable to phenomena such as the Hispanic health paradox, where strong community support may offset poor health outcomes [23]. Similarly, social networks can influence healthcare access—for example, limited transportation access can contribute to social isolation, thereby worsening frailty status and healthcare outcomes [24]. We observed this phenomenon as membership in the mildly and drastically worsening frailty trajectories was associated with higher odds of needing transportation assistance to the doctor and membership in the drastically worsening class was associated with lower odds of having a regular doctor. These findings reinforce the importance of social support as a modifiable factor in frailty prevention.

We anticipated that rural residence would be associated with higher frailty, SNF placement, and mortality. Surprisingly, there were no significant differences in these outcomes between rural and urban older adults. However, there were higher odds of SNF placement and death with increasing age, higher frailty counts, and worsening frailty trajectories, reaching statistical significance. Notably, female sex was associated with lower hazard of death, which supports the well-established sex-differences in health outcomes [25]. The lack of geographical effect may suggest that SDOH and health outcomes across rural and urban areas are more similar than previously assumed and or a limitation of the lack of standardized definitions of “rural” and “urban.” Even though NHATS is a representative sample of the United States, those with lower educational and income levels may still be underrepresented, limiting our generalizability. Future work will need to explore whether other factors of rural living such as potentially higher activity levels and household composition may contribute to similar health outcomes between rural and urban areas.

Our study is an innovative approach to investigating how SDOH may influence frailty status and trajectories, using latent class growth analysis. The results of our study have important clinical implications by identifying specific ecological characteristics that can be potentially addressed by both individual- and system-level supports and interventions. However, our study also presents various limitations. Because this was a secondary data analysis, we are unable to declare any causal associations. Additionally, Fried’s frailty phenotype scoring scale (0–5 points) may incur floor and ceiling effects and limit the applicability of our findings for individuals who fall on the extremes of this scale. Fried’s frailty phenotype also does not account for how cognitive changes impact frailty development and trajectory, which will be important to consider in future analyses as we observed higher percentages of older adults with dementia in the drastically worsening category (Table 1) [26].

We additionally acknowledge the limitation of RUCC to most accurately define “rural” and “urban” as the RUCC “non-metropolitan” and “metropolitan” classifications are not totally synonymous with “rural” and “urban,” respectively. Accurately defining residential status is a limitation of the current literature due to the complexity of definitions and inconsistent use in research. Due to the extensive NHATS database publications using RUCC codes, we used the same definitions to optimize comparability to previous findings, which is important for results reproducibility [14,15]. Future analyses will benefit from utilization of multiple definitions of “rural” and “urban” to best capture the characteristics of older adults in particular geographical areas.

It is important to note the smaller sample sizes of individuals identifying as Hispanic ethnicity, as this may have contributed to our observed findings reaching statistical significance. However, we feel that it is important to report this finding given the underrepresentation of this demographic in research including older adults. We feel that it is also important to note the structural bias in society that may impact accessibility to socioeconomic opportunities for certain race/ethnic groups. Lastly, while we included representative variables from all domains of SDOH (e.g., education, healthcare access, neighborhood, community, and income), this was not a fully exhaustive list and may miss important factors that influence healthcare delivery and frailty trajectories [24].

## 5. Conclusions

These findings substantiate the pervasive effect that SDOH (such as social support, transportation access, financial resources, education status) have in influencing the trajectory of frailty progression for older adults in urban and rural areas. Our analysis further highlights specific ecological factors that may be potential targets of interventions to address the differential healthcare needs of older adults in the United States with frailty.

## Figures and Tables

**Table 1.** Characteristics of study cohort by frailty trajectory classification using latent class growth analysis.

	Total (*n* = 6082)	Improving (*n* = 422)	Stable (*n* = 2396)	Mildly Worsening (*n* = 2518)	Drastically Worsening (*n* = 746)	
Variable	*n* (%)	*n* (%)	*n* (%)	*n* (%)	*n* (%)	*p*-Value
Demographics						
Urban Residence	4949 (82.0)	330 (77.1)	1967 (83.4)	2053 (81.7)	599 (80.7)	0.17
Age						
65–69 years	1154 (28.6)	119 (39.0)	582 (35.1)	369 (22.5)	84 (18.1)	<0.001
70–74 years	1274 (25.0)	109 (27.8)	565 (26.9)	484 (24.1)	116 (19.2)	
75–79 years	1230 (19.2)	70 (14.2)	445 (16.5)	533 (21.3)	182 (25.4)	
80–84 years	1203 (14.5)	66 (10.6)	411 (11.5)	543 (16.7)	183 (20.6)	
85–89 years	1221 (12.8)	58 (8.4)	393 (10.0)	589 (15.4)	181 (16.7)	
Sex						
Male	2529 (43.6)	172 (42.6)	1021 (44.8)	1048 (43.4)	288 (40.3)	0.34
Female	3553 (56.4)	250 (57.4)	1375 (55.2)	1470 (56.6)	458 (59.7)	
Race						
White	4194 (81.2)	269 (77.7)	1696 (83.2)	1722 (79.7)	507 (81.4)	0.11
Black	1318 (8.1)	110 (9.7)	491 (7.4)	547 (8.2)	170 (8.9)	
Hispanic	352 (6.5)	29 (8.1)	130 (5.8)	154 (7.3)	39 (5.3)	
Other	218 (4.2)	14 (4.4)	79 (3.6)	95 (4.8)	30 (4.4)	
Education						
Less than High School	1606 (20.8)	123 (22.1)	553 (17.4)	702 (23.0)	228 (25.0)	<0.001
High School	1642 (26.9)	111 (26.7)	597 (23.8)	727 (29.9)	207 (28.5)	
Some College	769 (13.9)	37 (9.7)	307 (13.8)	307 (13.9)	118 (17.7)	
College Degree and Beyond	2065 (38.4)	151 (41.5)	939 (45.0)	782 (33.3)	193 (28.9)	
Marital Status						
Married	2915 (54.6)	201 (56.2)	1237 (58.6)	1166 (52.3)	311 (46.5)	<0.001
Separated/Divorced	742 (12.3)	65 (15.8)	287 (11.6)	293 (12.1)	97 (14.0)	
Widowed	2061 (27.0)	127 (22.8)	720 (23.3)	915 (29.7)	299 (34.2)	
Never Married	239 (3.7)	20 (3.5)	97 (3.9)	94 (3.6)	28 (3.6)	
Living with Partner	125 (2.3)	9 (1.7)	55 (2.6)	50 (2.3)	11 (1.8)	
Income						
USD 0–24,999	1700 (24.7)	113 (22.6)	605 (20.8)	744 (27.5)	238 (30.7)	<0.001
USD 25,000–49,999	818 (14.1)	47 (12.4)	322 (13.8)	352 (15.2)	97 (12.4)	
USD 50,000–74,999	451 (8.7)	28 (7.7)	192 (8.9)	189 (9.2)	42 (6.9)	
USD 75,000–99,999	230 (4.9)	23 (7.2)	110 (6.1)	76 (3.7)	21 (3.7)	
USD 100,000–199,999	235 (5.3)	19 (6.6)	119 (6.9)	78 (3.8)	19 (3.4)	
USD 200,000+	85 (2.0)	7 (2.7)	50 (3.0)	22 (1.1)	6 (0.7)	
Missing	2563 (40.4)	185 (40.8)	998 (40.5)	1057 (39.6)	323 (42.2)	
Language						
Language other than English	1077 (19.6)	95 (26.4)	429 (19.7)	436 (19.2)	117 (16.2)	0.004
English	5005 (80.4)	327 (73.6)	1967 (80.3)	2082 (80.8)	629 (83.8)	
Insurance						
Medicaid	921 (11.7)	86 (15.4)	331 (10.0)	390 (12.4)	114 (13.0)	0.03
Medigap	3100 (54.1)	205 (53.6)	1217 (54.3)	1301 (53.9)	377 (54.6)	
Tricare	188 (3.3)	12 (3.7)	80 (3.5)	68 (2.8)	28 (4.1)	
Medicare only	1873 (30.9)	119 (27.3)	768 (32.2)	759 (30.9)	227 (28.3)	
Healthcare Access						
Has Regular Doctor	5796 (95.4)	403 (95.3)	2300 (96.0)	2390 (95.3)	703 (93.6)	0.24
Seen Doctor Last Year	5716 (93.6)	396 (93.9)	2257 (93.7)	2367 (93.6)	696 (92.7)	0.88
Community Support						
Household size	1.97 (0.02)	2.00 (0.05)	1.97 (0.02)	1.98 (0.03)	1.87 (0.04)	0.09
Need help in transportation to doctor	2189 (29.1)	152 (29.1)	752 (24.1)	974 (32.6)	311 (35.6)	<0.001
Health						
Cardiovascular Disease	1589 (24.2)	101 (20.5)	576 (21.8)	692 (26.3)	220 (28.2)	0.002
High Blood Pressure	4090 (63.8)	282 (61.3)	1564 (61.1)	1729 (66.6)	515 (65.9)	0.005
Arthritis	3399 (53.8)	249 (57.7)	1250 (49.4)	1459 (56.6)	441 (58.6)	<0.001
Osteoporosis	1252 (21.1)	92 (22.6)	470 (19.5)	526 (21.8)	164 (23.5)	0.14
Type II Diabetes	1527 (23.6)	100 (21.1)	568 (21.9)	667 (25.2)	192 (26.2)	0.02
Lung Disease	887 (14.7)	55 (12.1)	322 (13.1)	401 (17.0)	109 (14.3)	0.002
Stroke	698 (9.8)	56 (10.9)	231 (7.7)	306 (10.8)	105 (13.4)	<0.001
Dementia	308 (3.8)	17 (2.8)	90 (2.7)	147 (4.6)	54 (6.0)	<0.001
Cancer	1564 (25.7)	102 (24.3)	605 (24.7)	661 (26.6)	196 (27.3)	0.50
Multimorbidity	4490 (70.7)	308 (71.0)	1704 (67.0)	1907 (73.6)	571 (74.2)	<0.001

**Table 2.** Multinomial logistic regression results by frailty latent growth class.

	Response			
	Improving	Stable	Mildly Worsening	Drastically Worsening
Variable	OR (95% CI)	OR (95% CI)	OR (95% CI)	OR (95% CI)
Demographics				
Age				
65–69 years				
70–74 years	0.87 (0.64, 1.18)	1	1.28 (1.04, 1.58)	1.23 (0.86, 1.76)
75–79 years	0.71 (0.51, 1.00)	1	1.81 (1.49, 2.19)	2.63 (1.78, 3.89)
80–84 years	0.74 (0.52, 1.05)	1	2.03 (1.68, 2.44)	2.94 (2.14, 4.05)
85+ years	0.64 (0.40, 1.03)	1	2.08 (1.72, 2.53)	2.56 (1.74, 3.78)
Race				
White				
Black	1.27 (0.99, 1.63)	1	1.06 (0.93, 1.21)	1.09 (0.79, 1.50)
Hispanic	0.96 (0.46, 2.00)	1	1.29 (0.92, 1.80)	0.98 (0.58, 1.68)
Other	1.03 (0.47, 2.24)	1	1.60 (1.14, 2.24)	1.57 (0.81, 3.03)
Sex				
Male				
Female	1.06 (0.80, 1.41)	1	0.91 (0.79, 1.04)	0.93 (0.72, 1.20)
Education				
Less than High School				
High School	0.99 (0.70, 1.40)	1	1.08 (0.87, 1.35)	0.93 (0.74, 1.17)
Some College	0.61 (0.38, 1.00)	1	0.95 (0.74, 1.22)	1.12 (0.82, 1.53)

**Table 3.** Multinomial logistic regression results by frailty latent growth class.

	Response			
	Improving	Stable	Mildly Worsening	Drastically Worsening
Variable	OR (95% CI)	OR (95% CI)	OR (95% CI)	OR (95% CI)
College Degree and Beyond	0.79 (0.57, 1.10)	1	0.76 (0.63, 0.91)	0.64 (0.49, 0.84)
Marital Status				
Married				
Separated/Divorced	1.44 (0.91, 2.26)	1	1.13 (0.89, 1.43)	1.38 (0.98, 1.94)
Widowed	1.03 (0.71, 1.50)	1	1.06 (0.91, 1.25)	1.15 (0.88, 1.49)
Never Married	0.88 (0.40, 1.95)	1	0.99 (0.63, 1.57)	0.99 (0.66, 1.50)
Living with Partner	0.61 (0.26, 1.43)	1	1.03 (0.67, 1.57)	0.88 (0.40, 1.93)
Insurance				
Medicare				
Medicaid	1.55 (1.07, 2.24)	1	0.95 (0.77, 1.17)	1.00 (0.71, 1.41)
Medigap	1.27 (0.93, 1.74)	1	1.05 (0.90, 1.23)	1.17 (0.94, 1.46)
Tricare	1.39 (0.73, 2.65)	1	0.92 (0.63, 1.34)	1.50 (0.91, 2.48)
Medicare only				
Income				
USD 0–24,999				
USD 25,000–49,999	1.12 (0.66, 1.91)	1	0.95 (0.76, 1.18)	0.72 (0.51, 1.02)
USD 50,000–74,999	1.12 (0.62, 2.05)	1	1.02 (0.80, 1.31)	0.74 (0.52, 1.07)
USD 75,000–99,999	1.64 (0.91, 2.97)	1	0.67 (0.50, 0.90)	0.68 (0.39, 1.19)
USD 100,000–199,999	1.40 (0.78, 2.51)	1	0.65 (0.44, 0.96)	0.64 (0.36, 1.12)
USD 200,000+	1.18 (0.39, 3.56)	1	0.41 (0.27, 0.63)	0.28 (0.10, 0.78)
Missing	1.19 (0.86, 1.64)	1	0.80 (0.66, 0.98)	0.78 (0.61, 0.99)
Language				
Language other than English				
English	0.64 (0.46, 0.88)	1	1.18 (0.97, 1.43)	1.30 (0.90, 1.86)
Health				
Multimorbidity	1.18 (0.87, 1.62)	1	1.19 (1.02, 1.38)	1.15 (0.92, 1.43)
Healthcare Access				
Seen Doctor Last Year	1.04 (0.62, 1.76)	1	0.96 (0.75, 1.23)	0.88 (0.60, 1.29)
Has Regular Doctor	0.79 (0.36, 1.73)	1	0.81 (0.56, 1.18)	0.61 (0.38, 0.97)
Need help in transportation to doctor	1.24 (0.90, 1.69)	1	1.15 (1.01, 1.32)	1.22 (1.00, 1.48)
Geographical Residence				
Urban Residence	0.63 (0.40, 0.99)	1	0.92 (0.77, 1.10)	0.89 (0.66, 1.19)
Community				
Household size	1.00 (0.90, 1.12)	1	1.04 (0.97, 1.13)	0.97 (0.86, 1.11)

**Table 4.** Logistic regression on the incidence of nursing home placement (N = 6082).

Variable	OR (95/% CI)
Age	
65–69 years	
70–74 years	1.66 (0.94, 2.95)
75–79 years	2.21 (1.23, 3.98)
80–84 years	3.11 (1.84, 5.26)
85+ years	4.68 (2.85, 7.69)
Race	
White	
Black	0.79 (0.60, 1.05)
Hispanic	0.44 (0.22, 0.89)
Other	0.86 (0.38, 1.97)
Sex	
Male	
Female	1.42 (1.07, 1.90)
Education	
Less than High School	
High School	0.87 (0.64, 1.17)
Some College	1.14 (0.71, 1.83)
College Degree and Beyond	1.19 (0.83, 1.71)
Income	
USD 0–24,999	
USD 25,000–49,999	0.38 (0.23, 0.64)
USD 50,000–74,999	0.32 (0.16, 0.64)
USD 75,000–99,999	0.52 (0.23, 1.19)
USD 100,000–199,999	0.33 (0.14, 0.78)
USD 200,000+	0.52 (0.13, 2.08)
Missing	0.78 (0.58, 1.04)
Frailty Trajectory (latent class membership)	
Stable	
Improving	0.54 (0.17, 1.72)
Mildly worsening	1.09 (0.61, 1.95)
Drastically worsening	1.70 (0.77, 3.77)
Urban Residence	0.70 (0.40, 1.21)
Frailty Trajectory with Urban Interaction Term	
Stable	
Improving	1.49 (0.47, 4.73)
Mildly worsening	1.36 (0.71, 2.60)
Drastically worsening	1.48 (0.56, 3.88)
Frailty Count	1.51 (1.35, 1.69)

**Table 5.** Survival analysis of mortality (N = 6082).

Variable	Hazard Ratio	95% CI
Age		
65–69 years	-	-
70–74 years	1.49	(1.23, 1.80)
75–79 years	1.93	(1.61, 2.32)
80–84 years	3.01	(2.53, 3.59)
85+ years	5.29	(4.44, 6.29)
Sex		
Male	-	-
Female	0.66	(0.61, 0.72)
Race		
White	-	-
Black	1.01	(0.91, 1.12)
Hispanic	0.67	(0.55, 0.82)
Other	0.71	(0.54, 0.93)
Education		
Less than High School	-	-
High School	0.97	(0.87, 1.09)
Some College	1.04	(0.90, 1.20)
College Degree and Beyond	0.9	(0.80, 1.01)
Income		
USD 0–24,999	-	-
USD 25,000–49,999	0.93	(0.80, 1.07)
USD 50,000–74,999	0.86	(0.71, 1.04)
USD 75,000–99,999	0.72	(0.56, 0.93)
USD 100,000–199,999	0.69	(0.52, 0.91)
USD 200,000+	0.91	(0.56, 1.49)
Missing	1.04	(0.94, 1.14)
Frailty Trajectory (latent class membership)		
Improving	0.54	(0.36, 0.82)
Stable	-	-
Mildly worsening	1.7	(1.38, 2.09)
Drastically worsening	3.01	(2.30, 3.95)
Urban Residence	0.82	(0.68, 0.98)
Frailty Trajectory with Urban Interaction Term		
Improving	1.04	(0.66, 1.66)
Stable	-	-
Mildly worsening	1.12	(0.89, 1.41)
Drastically worsening	1.09	(0.81, 1.47)
Frailty count	1.69	(1.62, 1.76)

## Data Availability

Data is available upon request from the research team.

## Appendix A

**Table A1.** Baseline characteristics of demographic information for older adults within metropolitan and non-metropolitan areas in 2011.

	Total (*n* = 6082)	Metropolitan (*n* = 4949)	Non-Metro (*n* = 1133)	
Variable	*n* (%)	*n* (%)	*n* (%)	*p*-Value
Demographics				
Mean Age	75.12 (0.10)	75.16 (0.12)	74.94 (0.23)	0.41
Age				
65–69 years	1154 (28.6)	930 (28.7)	224 (27.7)	0.35
70–74 years	1274 (25.0)	1028 (24.6)	246 (26.8)	
75–79 years	1230 (19.2)	989 (19.0)	241 (19.9)	
80–84 years	1203 (14.5)	991 (14.6)	212 (13.8)	
85+ years	1221 (12.8)	1011 (13.0)	210 (11.8)	
Sex				
Male	2529 (43.6)	2019 (43.1)	510 (46.0)	0.11
Female	3553 (56.4)	2930 (56.9)	623 (54.0)	
Race				
White	4194 (81.2)	3263 (79.3)	931 (89.9)	0.14
Black	1318 (8.1)	1172 (8.7)	146 (5.0)	
Hispanic	352 (6.5)	318 (7.3)	34 (3.0)	
Other	218 (4.2)	196 (4.7)	22 (2.2)	
Education				
Less than High School	1582 (20.7)	1264 (20.2)	318 (23.1)	0.02
High School	1633 (27.0)	1271 (25.9)	362 (32.1)	
Some College	764 (13.9)	630 (13.9)	134 (14.0)	
College Degree and Beyond	2049 (38.3)	1732 (40.0)	317 (30.8)	
Marital Status				
Married	2914 (54.6)	2314 (53.8)	600 (58.2)	0.01
Separated/Divorced	740 (12.3)	635 (13.0)	105 (9.3)	
Widowed	2058 (27.0)	1685 (26.8)	373 (27.6)	
Never Married	239 (3.7)	210 (4.1)	29 (2.1)	
Living with Partner	125 (2.3)	99 (2.3)	26 (2.7)	
Residence Type				
Community	5753 (94.4)	4667 (94.0)	1086 (96.1)	0.03
Residential Care	329 (5.6)	282 (6.0)	47 (3.9)	
Income				
USD 0–24,999	1700 (24.7)	1360 (24.1)	340 (27.1)	0.02
USD 25,000–49,999	818 (14.1)	653 (13.7)	165 (15.6)	
USD 50,000–74,999	451 (8.7)	377 (8.9)	74 (8.1)	
USD 75,000–99,999	230 (4.9)	195 (5.1)	35 (4.0)	
USD 100,000–199,999	235 (5.3)	210 (5.8)	25 (3.0)	
USD 200,000+	85 (2.0)	79 (2.3)	6 (0.7)	
Missing	2563 (40.4)	2075 (40.1)	488 (41.6)	
Language				
Language other than English	886 (16.7)	787 (18.5)	99 (8.9)	0.002
English	4976 (83.3)	3969 (81.5)	1007 (91.1)	
Insurance				
Medicaid	921 (11.7)	745 (11.6)	176 (12.1)	0.01
Medigap	3100 (54.1)	2456 (52.9)	644 (59.8)	
Tricare	188 (3.3)	164 (3.6)	24 (2.0)	
Medicare only	1873 (30.9)	1584 (32.0)	289 (26.1)	
Healthcare Access				
Has Regular Doctor	5792 (95.4)	4731 (95.8)	1061 (93.8)	0.01
Seen Doctor Last Year	5708 (93.6)	4672 (94.2)	1036 (90.7)	0.003
Community Support				
Total hours help per month	47.06 (2.10)	47.63 (2.52)	44.38 (5.21)	0.60
Household size	1.97 (0.02)	1.98 (0.02)	1.90 (0.03)	0.04
Need help in transportation to doctor	2081 (29.8)	1716 (29.9)	365 (29.1)	0.74
Health				
Current Smoker	450 (15.4)	343 (14.1)	107 (21.1)	<0.001
Cardiovascular Disease	1589 (24.2)	1266 (23.6)	323 (26.9)	0.07
High Blood Pressure	4082 (63.8)	3356 (64.4)	726 (60.7)	0.04
Arthritis	3391 (53.8)	2732 (53.1)	659 (56.8)	0.08
Osteoporosis	1249 (21.1)	1030 (21.6)	219 (19.2)	0.07
Diabetes	1527 (23.6)	1260 (23.9)	267 (22.4)	0.31
Lung Disease	887 (14.7)	719 (14.6)	168 (14.9)	0.87
Stroke	697 (9.8)	545 (9.4)	152 (11.4)	0.07
Dementia	308 (3.8)	266 (4.1)	42 (2.7)	0.04
Cancer	1562 (25.7)	1288 (26.1)	274 (23.9)	0.18
Multimorbidity	4490 (70.7)	3663 (70.6)	827 (70.7)	0.98
Frailty				
Robust	1094 (23.0)	881 (23.1)	213 (22.8)	0.73
Pre-Frail	3242 (53.0)	2652 (53.3)	590 (51.9)	
Frail	1746 (24.0)	1416 (23.7)	330 (25.3)	

**Table A2.** Model selection for latent class growth analysis.

Degree	Latent Class	Loglik	AIC	BIC	SABIC	Entropy	ICL1	ICL2
1	2	−51,806.53	103,631.07	103,691.48	103,662.88	0.5824	105,451.83	105,832.83
1	3	−50,764.96	101,555.93	101,643.20	101,601.89	0.5728	104,497.74	105,081.80
1	4	−50,277.35	100,588.69	100,702.81	100,648.79	0.5935	104,130.57	104,869.58
1	5	−50,075.70	100,193.39	100,334.37	100,267.64	0.5261	104,973.36	106,023.83
1	6	−49,888.31	99,826.62	99,994.45	99,915.01	0.5213	105,211.34	106,664.46
2	2	−51,665.24	103,352.47	103,426.32	103,391.36	0.6110	105,066.31	105,351.99
2	3	−50,567.73	101,167.47	101,274.88	101,224.03	0.5997	103,949.67	104,395.96
2	4	−50,010.27	100,062.54	100,203.51	100,136.78	0.6211	103,397.94	103,999.48
2	5	−49,783.49	99,618.97	99,793.51	99,710.89	0.5559	104,140.50	105,005.20
2	6	−49,544.62	99,151.24	99,359.35	99,260.84	0.5523	104,238.34	105,380.25
3	2	−51,624.54	103,275.08	103,362.35	103,321.04	0.6237	104,948.61	105,182.78
3	3	−50,512.55	101,063.11	101,190.66	101,130.28	0.6094	103,800.22	104,194.73
3	4	−49,928.03	99,906.06	100,073.89	99,994.45	0.6328	103,169.95	103,700.50
3	5	−49,703.54	99,469.08	99,677.19	99,578.68	0.5647	103,938.50	104,737.70
3	6	−49,456.74	98,987.48	99,235.86	99,118.28	0.5637	103,990.45	105,011.03

AIC: Akaike Information Criterion; BIC: Bayesian Information Criterion; SABIC: Sample Size-Adjusted Bayesian Information Criterion; ICL1: Integrated Completed Likelihood 1; ICL2: Integrated Completed Likelihood 2.

**Table A3.** Machine learning prediction performance.

Method	Accuracy	Sensitivity	Precision	Specificity	f1	AUC
SVM	0.757	0.515	0.515	0.838	0.515	0.779 (0.774, 0.787)
Random Forest	0.726	0.451	0.451	0.817	0.451	0.768 (0.756, 0.781)
KNN	0.735	0.469	0.469	0.823	0.469	0.767 (0.754, 0.770)
XGBoost	0.722	0.444	0.444	0.815	0.444	0.740 (0.720, 0.760)

AUC: Area under the curve.

## Abbreviations

The following abbreviations are used in this manuscript:

SDOH: Social determinants of health
LCGA: Latent class growth analysis
SNF: Skilled nursing facility
